# *GT-GRN*: a graph transformer framework for enhanced gene regulatory network inference via multimodal embedding of expression data and existing network knowledge

**DOI:** 10.1093/bib/bbaf584

**Published:** 2025-11-09

**Authors:** Binon Teji, Swarup Roy, Dinabandhu Bhandari, Jugal Kalita

**Affiliations:** Network Reconstruction & Analysis (NetRA) Lab, Department of Computer Applications, Sikkim University, 6th Mile, Tadong 737102, Sikkim, India; Network Reconstruction & Analysis (NetRA) Lab, Department of Computer Applications, Sikkim University, 6th Mile, Tadong 737102, Sikkim, India; Department of Computer Science and Engineering, Tezpur University, Napaam, Tezpur 784028, Assam, India; Department of Computer Science and Engineering, Heritage Institute of Technology, Kolkata 700107, West Bengal, India; Department of Computer Science, University of Colorado, Colorado Springs, CO, 80918, United States

**Keywords:** network inference, graph transformer, graph generation, gene expression, single-cell RNA seq, microarray, data fusion, embedding, global embeddings

## Abstract

The inference of gene regulatory networks (GRNs) is critical for understanding the regulatory mechanisms underlying cellular development, functional specialization, and disease progression. Predicting regulatory gene interactions—often framed as a link prediction task—is a foundational step toward modeling cellular behavior. However, GRN inference from gene coexpression data alone is limited by noise, low interpretability, and difficulty in capturing indirect regulatory signals. Additionally, challenges such as data sparsity, nonlinearity, and complex gene interactions hinder accurate network reconstruction. To address these issues, we propose, a novel graph transformer (GT) based framework (*GT-GRN*) that enhances GRN inference by integrating multimodal gene embeddings. Our method combines three complementary sources of information: (i) autoencoder-based embeddings, which capture high-dimensional gene expression patterns while preserving biological signals; (ii) structural embeddings, derived from previously inferred GRNs and encoded via random walks and a Bidirectional Encoder Representations from Transformers (BERT) based language model to learn global gene representations; (iii) positional encodings, capturing each gene’s role within the network topology . These heterogeneous features are fused and processed using a GT, allowing the joint modeling of both local and global regulatory structures. Experimental results on benchmark datasets show that *GT-GRN* outperforms existing GRN inference methods in predictive accuracy and robustness. Furthermore, it reconstructs cell-type-specific GRNs with high fidelity and produces gene embeddings that generalize to other tasks such as cell-type annotation.

## Introduction

Systems Biology seeks to understand the big picture in the complex biological systems, focusing on the extraction of relevant biological information within an organism at the cellular level. Biological components, such as genes, interact with each other to reconstruct gene regulatory networks (GRNs) from observational gene expression data [1]. This process is used to unveil a complex web of interactions, shedding light on the underlying patterns that govern gene regulation. Inferring GRNs from gene expression data is crucial for understanding the molecular interaction patterns among genes. A gene network consists of interlinked genes, where the expression of a gene influences the activity of other genes in the network [2]. An effective approach to describing GRNs involves the use of graphical and mathematical modeling, often grounded in graph-theoretic formalism to capture complex interactions between genes. Formally, a GRN is represented as a network of nodes and edges, where the nodes represent genes, and edges represent the regulatory interactions between them [3]. GRN inference involves predicting the connections among macromolecules by analyzing their relative expression patterns.

Technologies such as DNA microarray [4], single-cell RNA sequencing (scRNA-seq) [5], and single-nucleus RNA sequencing (snRNA-seq) [6] have revolutionized transcriptomics by offering diverse and detailed insights into gene expression. Although each of these technologies has its unique strengths, they also come with certain limitations. Common limitations include noisy gene expression data, which often complicate the inference process. Furthermore, the dynamic and nonlinear nature of gene–gene regulatory interactions present a significant challenge, as traditional or linear methods often fail to capture the complex relationships comprehensively. In addition, GRNs tend to be sparse, further reducing the overall accuracy of inference methods. In the case of single-cell technologies, significant dropout events introduce a large number of zero counts in the expression matrix, adding another layer of complexity [7]. These challenges highlight the need for more robust and sophisticated approaches to effectively analyze and interpret gene expression data.

A wide range of supervised and unsupervised GRN inference methods has been developed to uncover the intricate relationships within gene networks [8, 9]. Early approaches relied on relatively simple techniques such as correlation analysis, mutual information (MI)-based methods, and differential equation models. Attempts have been made to understand these complex relationships [10–12]. However, many of these methods exhibit inherent limitations. Thus, the development of more reliable GRN inference techniques remains an important research goal, and numerous intelligent computational strategies have been proposed to address this challenge.

Despite these advancements notable limitations persist. A key concern is that many approaches rely solely on a single source of information, typically gene expression data, to infer GRNs. This alone is often insufficient for accurate and reliable network prediction. In addition, some methods fail to incorporate knowledge from previously inferred GRNs, restricting their ability to build on existing insights. Furthermore, several techniques overlook the integration of topological information, which is essential to capture structural properties critical to robust GRN inference.

Rather than focusing solely on each aforementioned issue, our approach adopts an integrated perspective driven by the intuition that combining multiple complementary sources of information, beyond gene expression alone, can enhance the quality of GRN inference. We propose GT-GRN, a novel approach that integrates the strengths of both unsupervised inference methods and supervised learning frameworks. Our method combines outcomes from the available inference techniques to minimize method-specific biases, ultimately deriving a more realistic and biologically meaningful GRN. To integrate multiple networks, rather than relying on GNN-based methods, which often suffer from over-smoothing when stacking multiple layers. We adopt a state-of-the-art unsupervised approach based on NLP that effectively captures and integrates information across networks. GT-GRN leverages the latest advancements in Graph Transformer models to enhance GRN inference. GT-GRN integrates three distinct representations derived from input expression networks: (i) topological features, which capture the structural properties of the network; (ii) gene expression values, which are crucial for identifying gene interactions; and (iii) the positional importance of genes, which reflects their functional relevance within the network. By fusing the multimodal embeddings from diverse perspectives, our framework improves both the interpretability and predictive power of inferred GRNs, making it a robust solution for various biological applications. GT-GRN superiority comes from multiple design decisions.

(1) *Multinetwork integration*: A key challenge in supervised GRN inference is the absence of ground-truth networks. True GRNs are often incomplete or unavailable, so we must rely on inferred networks as proxies. However, using a single inferred network can introduce bias or overlook critical interactions. We incorporate multiple networks inferred by different methods, harnessing their complementary strengths. While various inference models exist, each with its own set of advantages and limitations, combining these diverse sources allows us to leverage their shared strengths. This approach helps mitigate methodological bias, ultimately enhancing the confidence and accuracy of our GRN predictions.
(2) *Gene expression embedding*: Capturing meaningful representations of gene expression data through advanced embedding techniques can provide a richer understanding of the underlying regulatory mechanisms and improve GRN inference.
(3) *GT frameworks*: Traditional GNNs rely on local message-passing mechanisms to infer graph structures. However, adopting GT-based frameworks offers a more effective encoding strategy by leveraging global attention mechanisms, enabling better capture of complex regulatory relationships in GRNs.

The contributions of our present work are listed below:

- We capture the quantitative characteristics of gene expression profiles through an autoencoder that learns biologically meaningful latent representations, effectively summarizing complex gene activity patterns while preserving essential regulatory signals (Section “Gene Expression Feature Encoding” ).
- We introduce a method to consolidate prior knowledge from multiple inferred GRNs by converting networks into text-like sequences, enabling a BERT-based masked language model to learn global gene embeddings that integrate structural information across all networks (Section “Global Embeddings via multinetwork integration of the inferred GRNs”).
- We propose a novel framework, GT-GRN, which leverages attention mechanisms within a GT model to learn rich gene embeddings by integrating multisource data—including gene expression profiles, structured inferred knowledge, and graph positional encodings from the input graph. These unified gene embeddings effectively capture the underlying biological relationships between genes, facilitating enhanced GRN inference (Section “Graph transformer for GRN inference”).
- We demonstrate that GT-GRN effectively advances cell-type-specific GRN reconstruction. Moreover, the superior quality of the learned embeddings enables their successful application to cell type annotation tasks, highlighting the model’s robustness and generalizability.

The remainder of the paper is organized as follows. Section “Related work” reviews related work in GRN inference. Section “Materials and methodology” describes the proposed GT-GRN framework, including gene expression embedding, multinetwork integration, and graph positional encoding, along with details of the datasets used. Section “Results and analysis” presents experimental results and analysis. Section “Application of GT-GRN on cell-type classification” demonstrates the classification capabilities of GT-GRN. Finally, Section “Conclusion” concludes the paper by summarizing key findings and describing directions for future research.

## Related work

With decades of effort within the research community dedicated to deciphering gene regulatory relationships from gene expression data, numerous methods have been proposed for reconstructing GRNs [13]. Traditional approaches include regression-based techniques [14] and MI-based methods, such as Algorithm for the Reconstruction of Accurate Cellular Networks (ARACNE) [15], Minimum Redundancy Networks (MRNET) [16], and Context Likelihood of Relatedness (CLR) [17], which assess statistical dependencies between genes to infer potential regulatory interactions. Network Structure Controlling-based GRN inference method (NSCGRN) [18] is a global network partitioning and local network motif-based control framework for GRN inference. Global structure dominates the overall network structure to enforce hierarchy and sparsity while the local topology is refined using four known network motifs to adjust the specific patterns to improve biological plausibility.

Efforts from the area of computational biology have addressed data imbalance and noise with innovative modeling strategies based on complex-valued polynomial models [19]. Other approaches based on optimization, such as PGRNIG [20], which combines a parallel whale optimization algorithm with decomposition and regularization strategies, have shown high accuracy and speed in GRN inference from time-series data.

Supervised machine learning methods have also been explored for GRN inference. Support vector machines (SVMs) have been utilized to reconstruct biological networks through local modeling approaches [21]. Extensions such as CompareSVM [22] and GRADIS [23] further leverage classification-based frameworks to enhance network prediction accuracy.

With the advent of deep learning, more powerful and data-driven models have emerged. For instance, Daoudi *et al*. [24] proposed a deep neural network (DNN) model to infer GRNs from experimental data. Turki *et al.* [25] integrated both supervised and unsupervised learning techniques to perform link prediction on time-series gene expression data. Mao *et al.* [26] introduced a 3D convolutional neural network (CNN) model utilizing single-cell transcriptomic data, employing a novel labeling trick to enhance performance. GNE [27], a graph-based deep learning framework, unified known gene interactions, and expression profiles to robustly infer GRNs in a scalable manner. Other works such as Teji *et al.*, [28, 29], use synthetic data to evaluate various embedding models to evaluate for GRN inference in link prediction setup.

Significant progress has been made in developing approaches that incorporate machine learning techniques to infer the network from gene expression [30]. A recent trend in network-science has gained significant momentum in modeling graph-based applications powered by graph neural networks (GNNs). For example, Wang *et al.* propose GRGNN [31] method to reconstruct GRNs from gene expression data in a supervised and semi-supervised framework. The problem is formulated as a graph classification problem for GRN inference on DREAM5 benchmarks. Q-graph attention network (GAT) [32] proposes a quadratic complexity neuronal network using dual attention mechanism for GRN inference. The model is validated by introducing adversarial perturbations to the gene expression data on *E. coli* (Escherichia coli) and *S. cerevisiae* datasets. Huang *et al.* [33] propose a GNN-based model called MIGGRI for GRN inference using spatial expression images that capture gene regulation from multiple images. DeepRIG [34] emphasizes learning global regulatory structures by embedding entire graphs using a graph autoencoder, thereby capturing comprehensive latent representations. GMFGRN [35] applies graph convolutional networks (GCNs) to factorize scRNA-seq data into gene and cell embeddings, which are then used in a multilayer perceptron (MLP) for interaction prediction. GNNLink [36] frames GRN inference as a link prediction problem by employing a GCN-based interaction graph encoder to capture and infer potential regulatory dependencies between genes. AnomalGRN [37] addresses the challenges of heterogeneity and sparsity in GRNs by reformulating GRN inference as a node prediction task. To tackle the pronounced imbalance between positive and negative links, the authors cast the problem as a graph anomaly detection (GAD) task, enabling the identification of anomalous regulatory patterns within the network.

Another line of work also concentrates on Transformer-based architectures for GRN inference. TRENDY [38] leverages transformer models to construct a pseudo-covariance matrix as part of the WENDY [39] framework. Rather than generating GRNs from scratch, it enhances existing inferred GRNs. However, it does not incorporate additional structural side information into the inference process. STGRNS [40] is an interpretable transformer-based method for inferring GRNs from scRNA-seq data. This method only considers two genes for gene regulation excluding the possibility of indirect regulation for prediction. GRN-Transformer [41] utilizes multiple statistical features extracted from scRNA-seq data and uses inferred GRN extracted from a single inference algorithm PIDC [42].

Despite these advancements, many GRN inference methods still rely on a single source of data or even purely topological information. They often emphasize local or pairwise gene interactions. This narrow focus limits the depth and breadth of biological insights. Although deep learning approaches such as MLPs, CNNs, and GNNs have significantly improved inference performance, they frequently process each data modality in isolation, missing opportunities for deeper integration.

The present research takes a step forward by proposing a novel GT framework that integrates multiple sources of biological information for GRN inference. Unlike conventional methods that rely on convolution-based architectures, our approach leverages graph-based attention mechanisms to effectively model complex regulatory relationships. A key strength of this framework lies in its ability to fuse diverse embeddings derived from gene expression data, input graph structures, and both existing and previously inferred regulatory networks. By jointly leveraging these complementary sources within a unified model, it enables more accurate and biologically meaningful inference of GRNs.

## Materials and methodology

This section outlines the methodology of the proposed GT-GRN framework for GRN inference, followed by a description of the datasets used for evaluation. The framework is composed of three key modules: (i) encoding gene expression profiles as embedding features using unsupervised deep learning; (ii) extracting global gene embeddings through multinetwork integration; and (iii) capturing graph positional encodings from the input network structure. These complementary representations—gene expression embeddings, prior gene representations, and graph positional encodings—are fused to enhance GRN interaction prediction within a GT model. The effectiveness of our approach is then evaluated using publicly available gene expression datasets.

### Gene expression feature encoding

**Figure 1:**
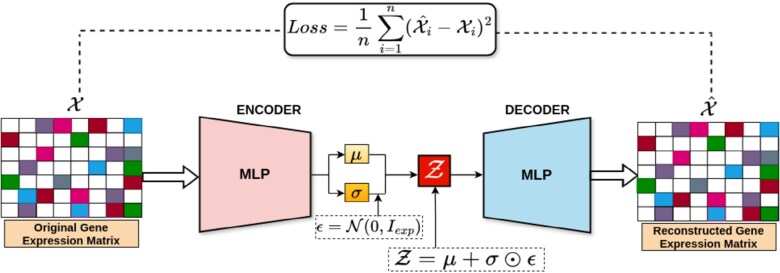
Variational autoencoder (VAE) for gene expression embedding, where the encoder maps the original gene expression matrix $\mathcal{X}$ into latent variables ($\mu,$$\sigma $), and the decoder reconstructs $\mathcal{X}$ from the latent representation $\mathcal{Z}$ using a neural network to minimize the reconstruction loss.

Gene expression data are increasingly complex due to advances in profiling technologies, making traditional linear models insufficient to capture its intricate patterns. Unsupervised deep learning, particularly variational autoencoders (VAEs) [43], offers a powerful way to nonlinearly encode such data into compact, informative representations that better reflect underlying biological structures. Figure 1 illustrates the overview VAEs for encoding gene expressions. VAEs are a probabilistic deep generative class of neural networks designed to reconstruct input data by learning a compressed, low-dimensional representation that effectively characterizes the input. Using VAE for gene expression encoding, we can efficiently capture complex expression dynamics and generate compact feature representations for further analysis and downstream tasks. VAEs are a powerful framework for unsupervised learning and generally comprise two interconnected components: an *encoder* and a *decoder*.

- Encoder: It maps the input gene expression matrix $\mathcal{X}$ to a latent representation space $\mathcal{Z}$. It approximates the posterior distribution $\alpha (\mathcal{Z}|\mathcal{X})$ using a neural network. The encoder outputs the parameters, i.e. the mean and variance of a multivariate Gaussian distribution $\beta (\mathcal{Z}|\mathcal{X})$, that serves as an approximation of the true posterior $\alpha (\mathcal{Z}|\mathcal{X})$. This process captures the underlying biological variability and regulatory patterns among genes.
- Decoder: It takes a sample $\mathcal{Z}$ from the latent space and maps it back to the original gene expression space, generating a reconstructed matrix $\hat{\mathcal{X}}$. This is modeled by the likelihood $\alpha (\mathcal{X}|\mathcal{Z})$, which represents the probability of generating the observed gene expression profiles $\mathcal{X}$ given the latent variables $\mathcal{Z}$. The decoder, implemented via a neural network, learns to reconstruct biologically plausible gene expression patterns from the learned latent representations.

During training, the VAE aims to learn the parameters of the encoder and the decoder network parameters by maximizing the Evidence Lower Bound (ELBO) which is given by: 


(1) \begin{align*}& \text{ELBO} = \mathbb{E}_{\beta(\mathcal{Z}|\mathcal{X})} [\log \alpha(\mathcal{X}|\mathcal{Z})] - \text{KL}[\beta(\mathcal{Z}|\mathcal{X}) || \alpha(\mathcal{Z})]\end{align*}

where $\mathbb{E}_{\beta (\mathcal{Z}|\mathcal{X})} [\log \alpha (\mathcal{X}|\mathcal{Z})]$ is the reconstruction term that reconstructs the input gene expression data $\mathcal{X}$ given the latent representation $\mathcal{Z}$. $\text{KL}[\beta (\mathcal{Z}|\mathcal{X}) || \alpha (\mathcal{Z})]$ is the KL (Kullback–Leibler) divergence that quantifies the distance between the approximate posterior $\beta (\mathcal{Z}|\mathcal{X})$ and the prior distribution $\alpha (\mathcal{Z})$.

Since sampling from learned distributions is inherently nondifferentiable, it hinders the use of gradient-based optimization during backpropagation. To address this, the reparameterization trick introduces a differentiable transformation by expressing the random variable as $\mathcal{Z} = \tau (\mathcal{X}, \epsilon )$, where, $\tau $ is a deterministic function and $\epsilon $ is an auxiliary noise variable drawn from a fixed, independent distribution. The above problem can be rewritten as: 


\begin{align*} & \mathcal{Z} \sim \beta(\mathcal{Z}|\mathcal{X}^{(i)}) = \mathcal{N}(\mathcal{Z} ; \mu^{(i)}, \sigma^{2(i)} I_{exp})\end{align*}

\begin{align*} &\mathcal{Z} = \mu + \sigma \odot \epsilon \text{; reparamterization trick}\end{align*}

Where $\epsilon = \mathcal{N}(0, I_{exp})$, $\odot $ is the element-wise product and $I_{exp}$ is the identity matrix, which serves as the covariance matrix.

### Global embeddings via multinetwork integration of the inferred gene regulatory networks

**Figure 2:**
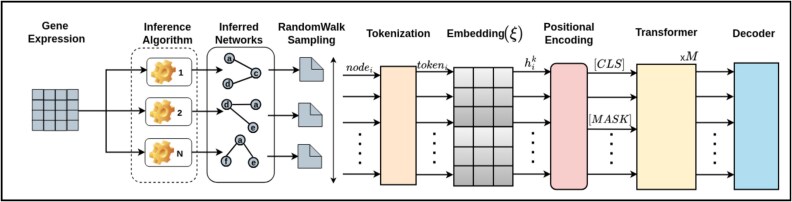
Global gene embeddings via multinetwork integration, where gene expression data are processed through inference algorithms to generate networks, sampled via random walks into node sequences beginning with a [CLS] token, tokenized and embedded, and passed through a transformer trained via masked node prediction to produce final gene embeddings.

We integrate multinetwork information to understand gene interactions as prior knowledge. Integrating data from diverse inference methods, each with unique strengths and limitations, provides a holistic and reliable view of the network. This approach overcomes the shortcomings of relying on a single method, enabling robust downstream analysis. Figure 2 illustrates the workflow.

#### Unsupervised network integration via random walks and transformers

We present an unsupervised learning approach for integrating multiple networks to generate global embeddings. Let a graph $\mathcal{G} = (\mathcal{V}, \mathcal{E})$ represent an inferred network, where $\mathcal{V}$ denotes the set of nodes and $\mathcal{E}$ represents the set of edges. The graph is characterized by its adjacency matrix $\mathcal{A} \in \mathbb{R}^{|\mathcal{V}| \times |\mathcal{V}|}$, where each entry $\mathcal{A}_{ij}$ reflects the relationship between nodes $v_{i}$ and $v_{j}$. Specifically, $\mathcal{A}_{ij} = 1$ if an edge $e_{v_{i}, v_{j}} \in \mathcal{E}$ connects $v_{i}$ and $v_{j}$, and $\mathcal{A}_{ij} = 0$ otherwise, indicating no direct connection.

We consider a collection of $C$ networks, $\{G_{1}, G_{2}, \dots , G_{C}\}$, sharing the same set of $n$ nodes but differing in the number of edges in each network. We capture the structural information of the networks by converting them into text-like sequences using random walks, similar to node2vec [44]. The walks are encoded through an embedding matrix $\xi \in \mathbb{R}^{n \times d_{n}}$, where $n$ is the size of the vocabulary (total nodes across all networks) and $p$ is the desired embedding dimension. Positional encodings are used to account for node order as described in [45]: 


(2) \begin{align*}& PE_{seq}{\text{(pos, i)}} = \begin{cases} \sin\left(\frac{\text{pos}}{10,000^{i/p}}\right)\!, & \text{if}\ i \mod 2 = 0, \\ \cos\left(\frac{\text{pos}}{10,000^{(i-1)/p}}\right)\!, & \text{otherwise}. \end{cases}\end{align*}

Here, $PE_{seq}{\text{(pos, i)}}$ represents the $i$th coordinate of the position encoding at sequence position $pos$. These encodings are concatenated with the original input features or the embedding matrix.

The embedding matrix and the decoding layer are initialized with uniform random values, while the transformer layer is initialized using Xavier’s initialization [46]. During training, all parameters are updated. Each sequence begins with a special classification token $[CLS]$, while other tokens correspond to node-specific vectors from the embedding matrix. The final hidden state of the $[CLS]$ token for a given sequence serves as the sequence representation.

#### Masked language learning with BERT

We utilize the Masked Language Modeling (MLM) approach as implemented in BERT [46]. At its core, this method employs a transformer encoder composed of $ M$ identical blocks. Each block includes a self-attention mechanism followed by a feedforward neural network (FFN), as described in [45].

Let $ F = [f_{1}, f_{2}, f_{3}, \dots , f_{n}] $ denotes an input sequence of $ n $ tokens, where each token is represented by a $ p $-dimensional vector. A self-attention layer processes this sequence using the following transformation: 


(3) \begin{align*}& \text{Attention}_{seq}(Q_{seq}, K_{seq}, V_{seq}) = \text{softmax}\left(\frac{{Q_{seq}}{K_{seq}}^\top}{\sqrt{{d_{n}}_{k_{seq}}}}\right)V_{seq},\end{align*}

where $ Q_{seq} = W_{q{\_}seq}F $, $ K_{seq} = W_{k{\_}seq}F $, and $ V_{seq} = W_{v{\_}seq}F $. Here, $ W_{q{\_}seq} $, $ W_{k{\_}seq} $, and $ W_{v{\_}seq} $ are learnable matrices that project the input into query, key, and value spaces of node sequences, respectively. ${d_{n}}_{k_{seq}}$ is the dimension of the key vectors.

The feedforward layer, applied independently to each token, performs the transformation: 


(4) \begin{align*}& FFN(\xi) = \text{ReLU}(\xi W_{seq1} + b_{seq1})W_{seq2} + b_{seq2},\end{align*}

where $ W_{seq1} $ and $ W_{seq2} $ are learnable matrices, and $ b_{seq1} $ and $ b_{seq2} $ are bias vectors. FFN is the feed-forward network and $\xi $ is the global gene embeddings.

The MLM task involves masking a random subset of input tokens and predicting their identities based on the remaining context. This encourages the model to capture bidirectional contextual relationships within sequences. Specifically, we mask $ 20\% $ of the tokens (representing nodes) in each sequence and train the model to recover the masked tokens using a cross-entropy loss function: 


(5) \begin{align*}& L_{BERT} = -\sum_{b=1}^{B} \sum_{ln=1}^{Ln} \sum_{cl=1}^{Mc} 1_{\{b, ln \in \text{mask}\}} \cdot y_{b,ln,cl} \log p_{b,ln,cl},\end{align*}

where $ B $ is the batch size, $ Ln $ is the sequence length and $ Mc $ is the number of classes (total number of possible tokens that can be predicted). $y_{b,ln,cl}$ is a binary indicator equal to $ 1 $ if the correct class of token $ ln $ in batch $ b $ is $ cl $, and $ p_{b,ln,cl} $ is the predicted probability for this classification. The final embeddings are extracted from the embedding layer represented as $\xi $.

This enables the model to learn rich contextual embeddings for nodes, capturing both structural and positional relationships within the networks.

### Graph transformer for gene regulatory network inference

**Figure 3:**
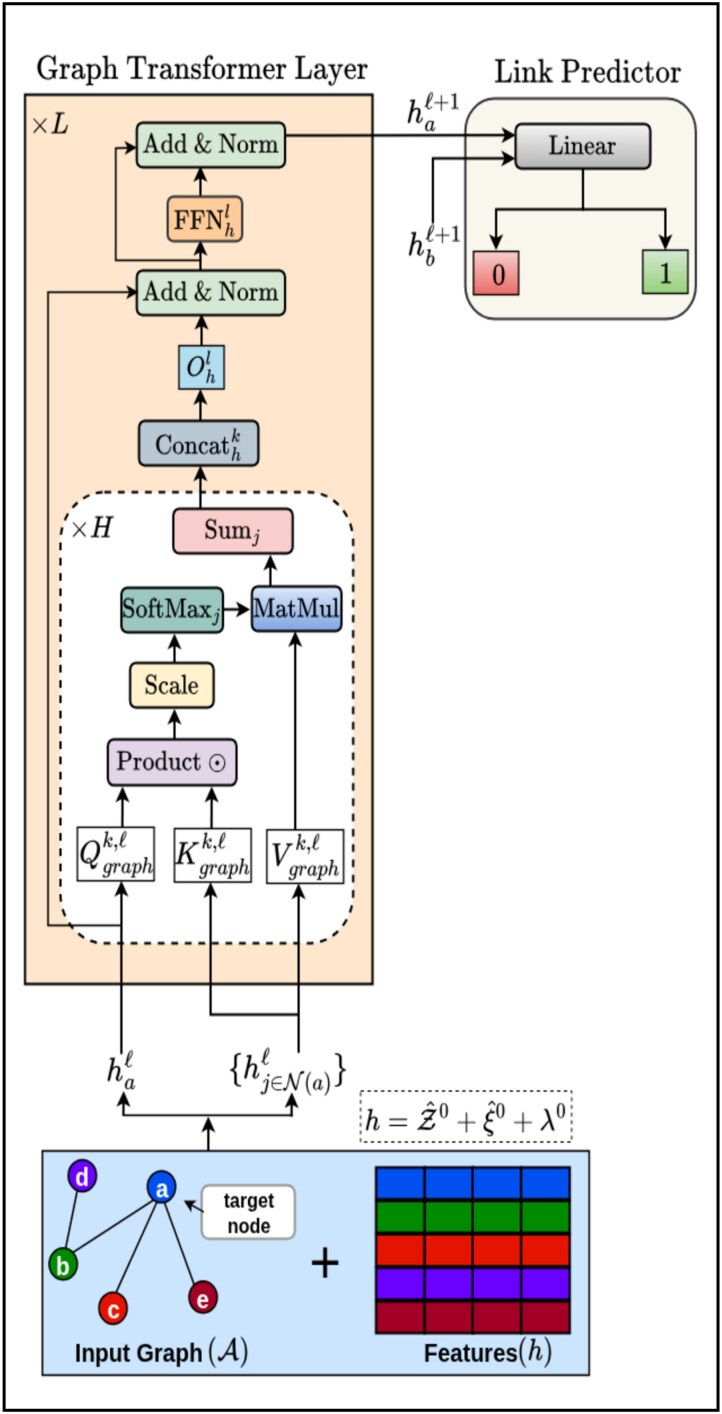
Architecture diagram of GT-GRN, where graph transformer layer processes input graph with gene expression, graph positional, and global embeddings to generate gene representations, followed by a link predictor module that estimates connections between two genes using their embeddings $h_{a}^{\ell +1}$ and $h_{b}^{\ell +1}$.

After deriving features from available GRNs and gene expression data, we utilize the GT to learn comprehensive representations by injecting the underlying regulatory structure. Since GT is specifically designed to model complex dependencies in graph-structured data, it effectively captures gene-gene interactions based on attention mechanisms, making it well suited for GRN-based representation learning. GT-GRN is illustrated in Fig. 3.

#### Graph positional encodings

NLP-oriented Transformers are supplied with Positional Encodings. At the heart of GT, graph positional encodings hold a special place which is important for encoding node positions. From the available graph structure, we make use of Laplacian eigenvectors and use them as graph positional encoding ($PE_{graph}$) information. This is helpful to encode distance-aware information, i.e. nearby nodes would have similar positional features and vice versa. Eigenvectors are defined as the factorization of the graph Laplacian matrix: 


(6) \begin{align*}& \Delta = I_{graph} - D^{-\frac{1}{2}} \mathcal{A} \thinspace D^{-\frac{1}{2}} = U \Lambda U^{T}\end{align*}

where $\mathcal{A}$ is the input adjacency matrix, $I_{graph}$ is the identity matrix of size $n \times n$. $D$ is the degree matrix, $\Lambda $ is the eigen-values and $U$ are the eigenvectors. We then use the $p$ smallest significant eigen-vectors of a node as its positional encoding, which is denoted by $\lambda _{i}$ for node $i$.

#### Input to GT-GRN 

The input to the GT layer is the graph structure $ \mathcal{G} $ and its associated features $ h $. The features $ h $ are constructed as a combination of gene expression embeddings ($ \mathcal{Z} $), global gene embeddings ($ \xi $), and graph positional embeddings ($ \lambda $), which are derived from the graph $ \mathcal{G} $.

- For gene expression embeddings, each gene $ \mathcal{Z}_{i} \in \mathbb{R}^{d_{n} \times 1} $ is passed through a linear projection layer to embed it into a $ d $-dimensional space.
  (7) \begin{align*}& \hat{\mathcal{Z}}^{0}_{i} = S^{0} \mathcal{Z}_{i} + s^{0}\end{align*}
  where, $ S^{0} \in \mathbb{R}^{d \times d_{n}} $ is a learnable weight matrix, $ s^{0} \in \mathbb{R}^{d} $ is the learnable bias vector, and $ \hat{\mathcal{Z}}^{0}_{i} \in \mathbb{R}^{d} $ is the projected embedding.
- For the global gene embeddings, each vector $ \xi _{i} \in \mathbb{R}^{d_{n} \times 1} $ is also embedded into a $d$-dimensional space using a separate linear projection layer. The transformation is defined as:
  (8) \begin{align*}& \hat{\xi}^{0}_{i} = T^{0} \xi_{i} + t^{0}\end{align*}
  where, $ T^{0} \in \mathbb{R}^{d \times d_{n}} $ is a learnable weight matrix, $ t^{0} \in \mathbb{R}^{d} $ is the learnable bias vector.
- The graph positional encodings are extracted from the input graph $\mathcal{G}$. For a particular gene’s positional encoding $ \lambda _{i} \in \mathbb{R}^{d_{n}} $ is embedded into $ d $-dimensional space using a linear projection layer which is given by:
  (9) \begin{align*}& \lambda^{0}_{i} = U^{0} \lambda_{i} + u^{0}\end{align*}
  where, $ U^{0} \in \mathbb{R}^{d \times d_{n}} $ is a learnable weight matrix, $ u^{0} \in \mathbb{R}^{d} $ is a learnable bias vector.

Finally, the gene expression embeddings $ \hat{\mathcal{Z}}^{0} \in \mathbb{R}^{n \times d} $, global gene embeddings $ \hat{\xi }^{0} \in \mathbb{R}^{n \times d} $, and graph positional embeddings $ \lambda ^{0} \in \mathbb{R}^{n \times d} $ are each projected into a shared $ d $-dimensional space through separate linear transformation layers. These projected representations are then summed element-wise (often called fusion by summation) to form the final node features $ h \in \mathbb{R}^{n \times d} $: 


(10) \begin{align*}& h = \hat{\mathcal{Z}}^{0} + \hat{\xi}^{0} + \lambda^{0}\end{align*}

where, $h$ is the unified, per-gene embedding that fuses its expression profile, its topological position in the regulatory network, and a dataset-wide global context. This final representation $ h $ is then injected into the GT layer along with the adjacency matrix $ \mathcal{A} $ of the input graph $ \mathcal{G} $.

#### Graph transformer layer

The node update in the GT at layer ${\ell }$ is defined as follows: 


(11) \begin{align*} & \hat{h}_{i}^{\ell+1} = O_{h}^{\ell} \, \bigg\|_{k=1}^{H} \left( \sum_{j \in \mathcal{N}(i)} w_{ij}^{k,\ell} V_{graph}^{k,\ell} h_{j}^{\ell} \right) \end{align*}

(12) \begin{align*} & \text{where} \ \ w_{ij}^{k,\ell} = \text{softmax}_{j} \left( \frac{Q_{graph}^{k,\ell}h_{i}^{\ell} \cdot K_{graph}^{k,\ell} h_{j}^{\ell}}{\sqrt{d_{k}}} \right)\!, \end{align*}

and $d_{k} = d/H$, $k=1 \ \ \text{to} \ \ H$ denotes number of attention heads, and $\big \|$ denotes the concatenation of the number of heads. $Q_{graph}^{k,{\ell }}$, $K_{graph}^{k,{\ell }}$, $V_{graph}^{k,{\ell }} \in \mathbb{R}^{d_{k} \times d}$, $O_{h}^{\ell } \in \mathbb{R}^{d \times d} $, The attention outputs $\hat{h}_{i}^{{\ell }+1}$ are then passed to an FFN preceded and succeeded by residual connections and normalization layers as: 


(13) \begin{align*} & \hat{\hat{h}}_{i}^{\ell+1} = \text{Norm} \left( h_{i}^{\ell} + \hat{h}_{i}^{\ell+1} \right) \end{align*}

(14) \begin{align*} & \hat{\hat{\hat{h}}}_{i}^{\ell+1} = W_{graph2}^{\ell} \, \text{ReLU} \left( W_{graph1}^{\ell} \hat{\hat{h}}_{i}^{\ell+1} \right) \end{align*}

(15) \begin{align*} & h_{i}^{\ell+1} = \text{Norm} \left( \hat{\hat{h}}_{i}^{\ell} + \hat{\hat{\hat{h}}}_{i}^{\ell+1} \right) \end{align*}

where $W_{graph1}^{\ell } \in \mathbb{R}^{2d \times d}$, $W_{graph2}^{\ell } \in \mathbb{R}^{d \times 2d}$, and $\hat{\hat{h}}_{i}^{{\ell }+ 1}$, $\hat{\hat{\hat{h}}}_{i}^{\ell + 1}$ are the intermediate representations. Norm could be either Layer-Norm [47] or BatchNorm [48].

#### Link prediction with learned representations

The final module is designed to predict edges between nodes using the learned node representations $ h^{\ell +1} $ obtained from the GT layer. The module takes as input the embeddings $ h^{\ell +1} \in \mathbb{R}^{n \times d} $, where $ n $ is the number of nodes and $ d $ is the embedding dimension, along with an edge index representing node pairs. For each edge $ (i, j) $, the embeddings of the source node $ h^{\ell +1}_{i} $ and the destination node $ h^{\ell +1}_{j} $ are extracted and concatenated to form a feature vector. This vector is passed through a decoder network consisting of a multilayer perceptron (MLP) with a hidden layer, ReLU activation, and an output layer, which reduces the concatenated vector to a scalar. The scalar represents the predicted likelihood of an edge between the nodes $ i $ and $ j $. By utilizing the updated node embeddings $ h^{\ell +1} $, this module effectively learns to identify and score potential edges in the graph.

Next, we discuss the experimental setup used to demonstrate the superiority of *GT-GRN.*

### Experimental setup

The performance of GT-GRN is evaluated on Linux based NVIDIA RTX A3000 GPU as the computing machine. The deep learning libraries used here are pytorch(https://pytorch.org/), dgl (https://www.dgl.ai/), scikit-learn (https://scikit-learn.org/stable/), Pytorch Geometric (https://pytorch-geometric.readthedocs.io/en/latest/index.html∖#).

### Datasets

We establish our findings on two scRNA-seq human cell types: human embryonic stem cells (hESC) [49] and mouse embryonic stem cells (mESC) [50] from the BEELINE [51] study. The cell-type-specific ChIP-seq ground-truth networks are used as a reference for these datasets. Additionally, we use the synthetic expression profiles generated using GeneNetWeaver (GNW) [52], a simulation tool developed for DREAM (Dialogue on Reverse Engineering Assessment and Methods) along with their corresponding ground-truth networks. The details of the datasets have been discussed in Table 1.

**Table 1:** Dataset statistics

Species/cell types	Type	Source	$\mathcal{V}$	$\mathcal{E}$
Yeast	Microarray	GNW	4000	11,323
hESC-500	scRNA-seq	BEELINE	910	3940
mESC-500	scRNA-seq	BEELINE	1120	20,923
hESC-1000	scRNA-seq	BEELINE	1410	6139
mESC-1000	scRNA-seq	BEELINE	1620	30,254

#### Preprocessing of raw data

We preprocess the raw scRNA-seq data using an established method [51] to handle redundancy. We filter out low-expressed genes and prioritized the variable ones. Primarily, the genes expressed in <10% of cells were removed. Then, we computed the variance and $P$-values for each gene, selecting those with *P*-values below.01 after Bonferroni correction. Gene expression levels were log-transformed for normalization. This yielded a feature matrix $ \mathcal{X} \in \mathbb{R}^{n \times m} $, where $ n $ is the number of genes and $ m $ is the number of cells. Furthermore, we adopt the approach of Pratapa *et al.* [51] to assess performance across different network sizes. Specifically, we rank genes by variance and select the most variable transcription factors (TFs), along with the top 500 and 1000 genes with the highest variability.

### Baseline methods

We evaluate the efficacy of GT-GRN against the existing baselines methods commonly used for inferring GRNs are shown in Table 2.

**Table 2:** Summary of GRN inference methods classified by category

Category	Method	Description
Graph neural network	GNNLink [36]^a^	Uses a GCN-based interaction graph encoder to capture gene expression patterns.
	GENELink [53]^b^	Leverages a GAT to infer GRNs via attention mechanisms.
	GNE [27]^c^	Uses an MLP to encode gene expression profiles and network topology for predicting gene regulatory links.
MI	ARACNE [15]^d^	Infers networks based on adaptive partitioning (AP) and MI.
	BC3NET [54]^e^	An ensemble technique derived from the C3NET algorithm employing bagging.
	C3MTC [55]^f^	Infers networks where edge weights are defined by MI values.
	C3NET [56]^g^	Uses MI and a maximization step to capture causal structure.
Feature selection	MRNET [16]^h^	Applies supervised gene selection using MRMR (maximum relevance/minimum redundancy).
Ensemble tree-based	GRNBOOST2 [57]^i^	A fast inference algorithm using stochastic gradient boosting regression.
	GENIE3 [58]^i^	A classic inference algorithm using random forest or extra trees regression.

^a^ https://github.com/sdesignates/GNNLink  ^b^https://github.com/zpliulab/GENELink  ^c^https://github.com/kckishan/GNE  ^d^https://bioconductor.org/packages/release/bioc/html/minet.html  ^e^https://cran.r-project.org/web/packages/bc3net/index.html  ^f^https://cran.r-project.org/web/packages/bc3net/index.html  ^g^https://cran.r-project.org/web/packages/c3net/index.html  ^h^https://bioconductor.org/packages/release/bioc/html/minet.html  ^i^https://github.com/aertslab/arboreto  ^j^https://github.com/aertslab/arboreto

## Results and Discussion

We report results using both single-cell and microarray gene expression datasets, selecting representative methods from each major category of GRN inference techniques against GT-GRN. These include MI-based methods, feature selection approaches, ensemble tree-based models, and graph neural network frameworks. This diverse selection allows us to comprehensively evaluate performance across different inference paradigms, ensuring a balanced comparison that highlights the strengths and limitations of each method.

### Gene regulatory network inference via full network reconstruction

**Figure 4:**
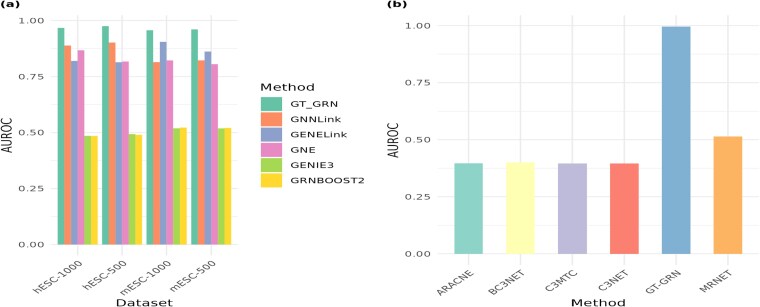
Full network reconstruction performance of various methods for different datasets in terms AUROC score. *(a) BEELINE’s scRNA-seq datasets and (b) GNW’s Yeast dataset.*

Fundamentally, GRN inference aims to reconstruct the entire regulatory network, capturing the full complexity of gene interactions. By striving for complete network reconstruction, it seeks to reveal the intricate web of regulatory relationships among all involved genes, reflecting the true, comprehensive regulatory architecture [59]. We evaluate the effectiveness of GT-GRN alongside existing methods designed for GRN inference. Following a similar motivation, we report these results for GT-GRN and its baseline methods in Fig. 4. In the figure, the results of the full network reconstruction, highlight the comparative performance of different methods. For the scRNA dataset, the performance of GT-GRN remains consistently higher with minor variations for all datasets. This suggests that the GT-GRN method is robust for different cell types and sequencing depths.

For the *Yeast* (microarray) dataset, the GT-GRN method significantly outperforms all other network inference methods, indicating its superior ability to capture gene interactions. Baseline methods such as ARACNE, BC3NET, C3MTC, C3NET, and MRNET perform at similar levels. In general, GT-GRN appears to be the most effective method for the microarray dataset, while for scRNA expression profiles the method demonstrate stable performance across conditions in terms of AUROC score.

### Gene regulatory network inference via link prediction

There exist many benchmark methods that treat GRN inference as a link prediction problem, focusing on identifying only a limited subset of interactions. We report results using this conventional approach. Table 3 reports the performance results for the candidate datasets in terms of *Area Under the Receiver-Operating Characteristic Curve* (AUROC) and *Area Under the Precision-Recall Curve* (AUPRC) metrics. To assess the predictive performance of GT-GRN, we present a comparison with various baseline methods. In the table it is clearly evident that the GT-GRN consistently outperforms other methods in all datasets, achieving the highest AUROC and excelling in AUPRC, particularly for the mESC-1000 and mESC-500 datasets. GNE emerges as a strong contender, especially in the hESC-1000 dataset, where it achieves the highest AUPRC $0.9042$ and the AUROC of $0.9025$, indicating its effectiveness in balancing precision and recall. GENELink maintains strong performance across most datasets, while GNNLink performs well in AUROC but lags in AUPRC. In contrast, GENIE3 and GRNBOOST2 show consistently lower scores, indicating challenges in handling these complex datasets. Notably, GNE outperforms all methods in both AUROC and AUPRC for the hESC-1000 dataset, highlighting its scalability. For the Yeast-4000 dataset, GT-GRN demonstrates superior performance compared to its baseline counterparts. Overall, GT-GRN proves to be the most reliable across datasets, while GNE stands out for its precision, making them the top choices for biological network predictions in this study.

**Table 3:** AUROC and AUPRC scores for different methods across the various scRNA-seq datasets

Dataset	Method	AUROC	AUPRC
mESC-1000	GT-GRN	**0.9483**	**0.8990**
	GNNLink	0.8833	0.8660
	GENELink	0.9133	0.8103
	GNE	0.8984	0.8925
mESC-500	GT-GRN	**0.9402**	**0.8853**
	GNNLink	0.8768	0.8331
	GENELink	0.9057	0.8004
	GNE	0.8378	0.8416
hESC-500	GT-GRN	**0.8793**	0.5932
	GNNLink	0.8251	0.4542
	GENELink	0.8618	0.5581
	GNE	0.8402	**0.8466**
hESC-1000	GT-GRN	0.8784	0.8604
	GNNLink	0.8442	0.5011
	GENELink	0.8657	0.5610
	GNE	**0.9025**	**0.9042**

Bold values indicate the best performance.

### Impact of hyperparameters

**Figure 5:**
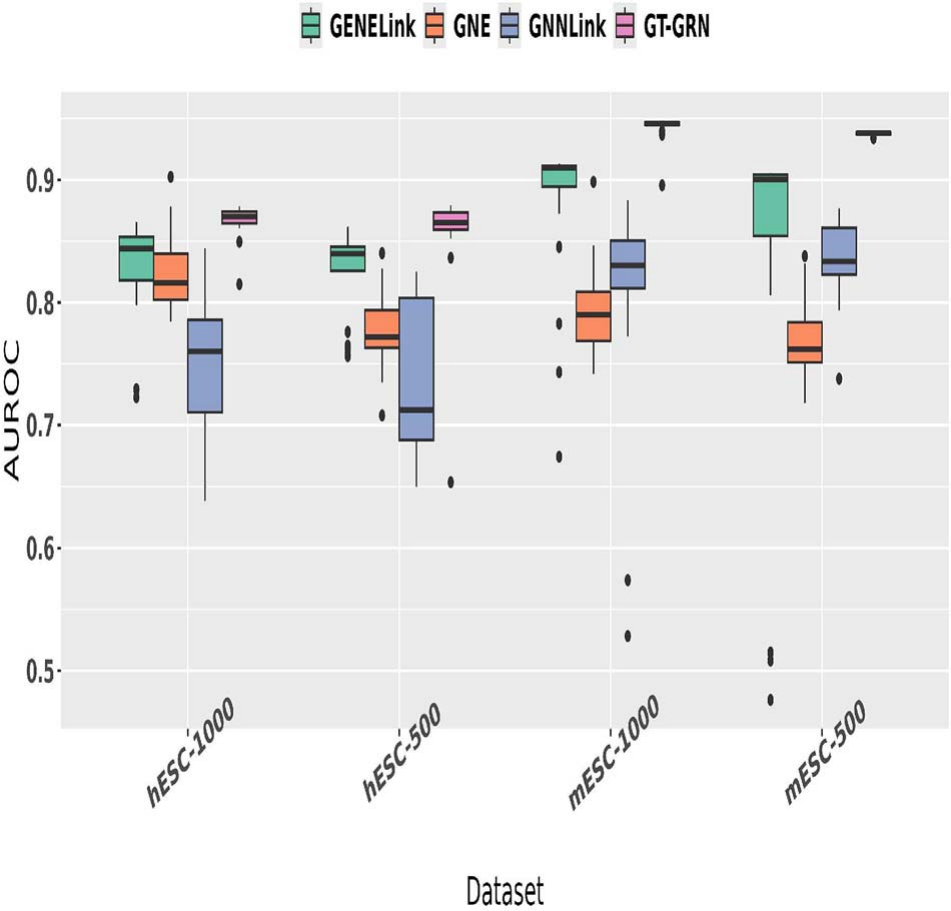
Overall HP tuning plot for various models.

Additionally, we investigate the role of various hyperparameters (HPs) that influence the overall performance of the models. Optimal selection of HPs is a difficult task and time-consuming activity. We analyze the behavior of the learning models compared to GT-GRN by selecting key HPs from each baseline model to assess their impact on the overall performance of each model. Table 4 describes the parametric configurations that we tuned for each learning model with their respective explanations. We summarize the overall impact of HP tuning for different models using a boxplot, as shown in Fig. 5. This visualization presents a comprehensive comparison of our model, GT-GRN, against several baseline approaches in terms of AUROC performance across multiple datasets.

**Table 4:** Tuned hyperparameters (HPs) for different models, where an *epoch* represents one full pass over the dataset, the *learning rate* controls the update pace, the *output dimension* defines the final prediction layer shape, *attention heads* compute and aggregate attention over input elements, and *layers* indicate the number of transformation steps applied sequentially to produce the output and pass to the next layer

Model	Epochs	Learning rate	Output dimensions	Attention heads	Layers
GT-GRN	–	0.001, 0.003, 0.0005	–	2,4,8	4,6,8
GNNLink	100, 200, 300	0.001, 0.005, 0.01	128, 256, 512	–	–
GENELink	10, 20, 30	0.001, 0.003, 0.0005	128, 256, 512	–	–
GNE	10, 20, 30	0.001, 0.005, 0.01	128, 256, 512	–	–

A key observation is that GT-GRN consistently achieves higher median AUROC scores with notably lower variance across datasets, highlighting its robustness and reliability under varying experimental conditions. Each model was tuned with its respective optimal HPs, yet GT-GRN exhibits both stability and effectiveness across settings, clearly outperforming the existing baselines on all candidate datasets. In contrast, GNNLink shows a higher variance, particularly in the hESC-500 dataset, where its performance fluctuates significantly. GENELink and GNE display relatively stable performances, though both fall short of the superior AUROC achieved by GT-GRN. In particular, the mESC-1000 dataset highlights the clear dominance of GT-GRN, with its AUROC surpassing that of all other methods by a substantial margin.

## Application of GT-GRN on cell-type classification

Cell-type-specific GRNs are crucial for defining transcriptional states during development, with each cell-type being characterized by a unique set of active TFs. These GRNs offer an unbiased method for studying gene regulation, providing valuable insights into the mechanisms driving cellular diversity. In this context, we explore the effectiveness of GT-GRN in reconstructing cell-type-specific GRNs, with the goal of cell-type annotation. To achieve this, we apply GT-GRN to scRNA-seq data from over 8000 human peripheral blood mononuclear cells (PBMCs8k), sourced from 10X Genomics (https://www.10xgenomics.com/datasets/8-k-pbm-cs-from-a-healthy-donor-2-standard-2-1-0). The data are preprocessed using the Scanpy framework [60], ensuring efficient handling and analysis of the single-cell data. For the ground truth network, we utilize the hTFtarget database [61], which integrates ChIP-seq data, TF binding sites, and epigenetic modification information. This comprehensive resource provides detailed insights into gene regulation and TF-target interactions, making it an invaluable tool for studying gene regulatory mechanisms.

In order to perform cell-type annotation, it is essential to first reconstruct the GRN. This involves generating embeddings that represent the cell types based on their GRNs. These embeddings serve as the foundation for cell-type classification, enabling accurate annotation of the cell types based on their unique regulatory patterns. By leveraging the power of GT-GRN in inferring cell-type-specific GRNs, we aim to advance the cell-type annotation in scRNA-seq data, ultimately improving our understanding of the complex regulatory landscapes that define cellular identities.

### GT-GRN for PBMC network reconstruction

We investigate GRN inference using GT-GRN by formulating it as a network regeneration problem on the PBMC dataset. First, we filter the data by removing genes expressed in <5% of cells and discarding cells that express <200 genes. Next, we normalize the total counts per cell to 10,000, ensuring comparability across cells. To further refine the data, we apply MAGIC [62] imputation, which reduces noise and improves expression patterns. Finally, we perform a logarithmic transformation to improve interpretability and optimize the data for downstream analysis.

After preprocessing, we employ GT-GRN to reconstruct the PBMC GRN and compare its performance against existing baseline methods. Comparison of PBMC’s hTFTarget (Gold standard) and generated networks in Table 5 reveals key structural differences and predictive performance variations. GT-GRN achieves the highest AUROC (0.9852) while maintaining balanced connectivity and clustering, making it the best-performing model. Supplementary Fig. S1 reports the ROC curve of the GT-GRN model showing high discriminative ability with performance well above the random baseline (dashed line). GENELink and GNNLink exhibit dense connectivity, high clustering, and shorter path length but are highly disassortative, indicating a strong preference for high-degree nodes connecting to low-degree ones. GNE, with lower connectivity and clustering, results in longer path lengths and the lowest AUROC (0.7596) but retains some structural similarities to the Gold network. The Gold standard itself maintains moderate connectivity and a sparse clustering structure, serving as a key benchmark. We also measure the quality of the generated graph network characteristics from the candidate methods with the input network (hTFTarget) using a single measurement score using Pearson correlation coefficient. The results show that all methods exhibit strong correlation, with GNE (0.9992) achieving the highest agreement, followed closely by GT-GRN (0.9838). However, GNNLink and GENELink report the similar score of 0.9817. These insights highlight the trade-offs between network structure and predictive performance, guiding model selection for biological network analysis.

**Table 5:** Network Characteristics Comparison of PBMC’s hTFTarget and generated networks with AUROC Score. *Maximum degree* computes the degree over all vertices. *Assortativity* is the Pearson correlation of degrees of connected nodes. *Triangle count* denotes the connection between two nodes. *Clustering coefficient* measure of the tendency of nodes in a network to form triangles. *Characteristic path length* represents the average shortest path length between all nodes pairs in a network. *PCC* is the Pearson Correlation Coefficient between hTFTarget and generated network characterisitics

Model	Maximum degree	Assortativity	Triangle count	Clustering coefficient	Characteristic path length	AUROC	PCC
Gold	1837	−0.4762	9596	0.0060	2.7392	–	–
GT-GRN	2593	−0.6614	207,579	0.0255	2.1934	0.9852	0.9838
GENELink	3999	−0.9867	5,671,530	0.0389	1.9731	0.8810	0.9817
GNNLink	3999	−0.9867	5,671,530	0.0389	1.9731	0.8467	0.9817
GNE	924	−0.1872	4014	0.0094	2.9621	0.7596	0.9992

**Figure 6:**
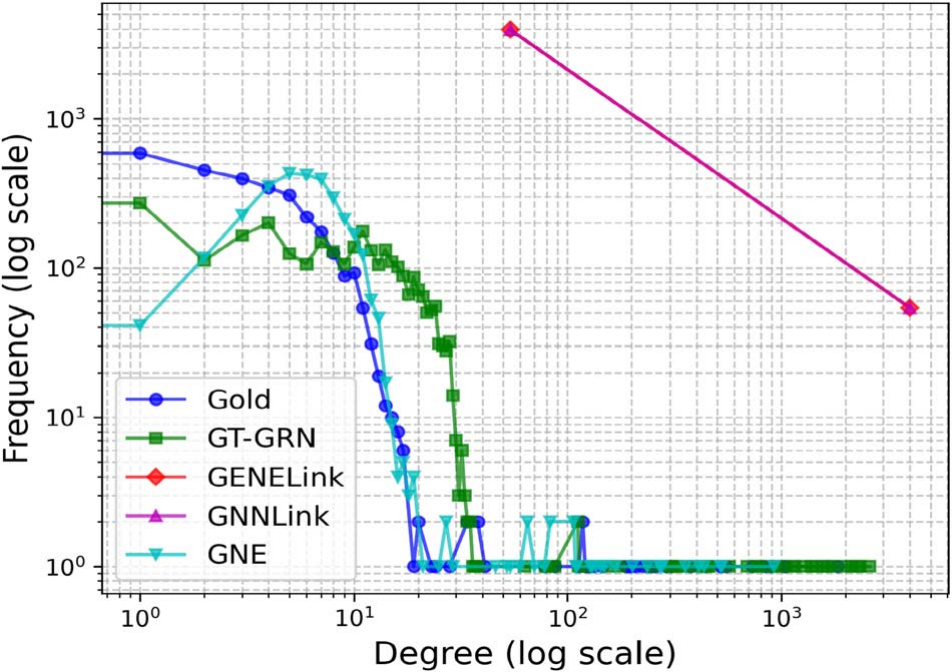
Log–log degree distribution plot of the input PBMC’s hTFTarget network and generated network for different models.

Further, we analyze the degree-distribution plot of the generated networks in comparison to the input PBMC’s hTFTarget network. Figure 6 describes the log–log degree distribution plot compares the degree distributions of the input (Gold) and generated networks (GT-GRN, GENELink, GNNLink, and GNE). The Gold network follows a natural decay which is scale-free in degree distribution, while GT-GRN shows a similar trend with slight deviations. GNE displays a more scattered pattern, indicating the similar tailed degree-distribution. GENELink and GNNLink exhibit significantly higher maximum degrees, i.e. these generated networks generate more high-degree nodes than the input network.

To optimize the architecture of GT-GRN model, we conducted a comprehensive HP search across different numbers of layers and attention heads on PBMC dataset. We evaluated model performance using the AUROC metric across varying combinations of input modalities. Positional embeddings, global and gene expression embeddings (unimodal), all pairwise combinations (bimodal), and the full trimodal input. Supplementary Fig. S2 shows the AUROC performance for each configuration focusing on the number of layers and attention heads. Each line represents a different head configuration (2, 4, and 8). For single-modal embeddings (top row), performance varies moderately with layer depth: positional embeddings show a decline at higher layers, whereas global and gene expression embeddings remain relatively stable. In two-modal combinations (middle row), AUROC is generally higher than in single-modal cases, indicating that combining modalities improves predictive performance. Some combinations benefit from deeper layers, while others peak at intermediate depths. For the three-modal combination (bottom row), integrating all three embeddings achieves the highest AUROC overall, although the optimal layer and head configuration differ slightly, reflecting complex interactions between modalities.

Furthermore, we report the computational efficiency of GT-GRN in comparison to the baselines on PBMC dataset, as detailed in Supplementary Table T1. It highlights a clear trade-off between predictive performance and resource requirements. GT-GRN incurs a higher computational cost compared with lightweight methods such as GNNLink and GNE, requiring $\sim $1.5 h for execution on the PBMC dataset, while GNNLink and GNE complete in under 12 min and 1 min, respectively. Although GT-GRN is more resource-intensive, this additional cost stems from its GT architecture, which jointly integrates positional, global, and gene expression embeddings. In contrast, baseline methods rely on simpler architectures with limited feature integration, resulting in faster runtimes but reduced representational capacity. Importantly, GT-GRN remains significantly more efficient than GENELink, which exceeds 2 h of runtime, suggesting that our method balances accuracy and computational feasibility.

**Figure 7:**
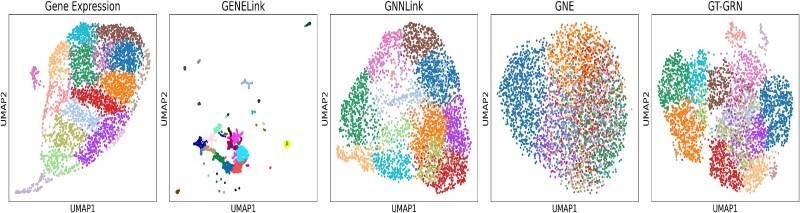
UMAP visualization of genes representations for PBMC’s dataset according to different methods.

Next, we assess how well our embeddings capture the community structure by clustering gene embeddings. We utilize the Leiden algorithm [63] to cluster the resultant gene embeddings. Figure 7 presents a uniform manifold approximation and projection (UMAP) dimensional reduction of gene representations for various methods. The methods compared include Gene Expression data, GENELink, GNNLink, GNE, and GT-GRN. The visualization clearly shows the distinct community structures produced by GNNLink, and GT-GRN embeddings that indicates effective preservation of biological modules. Notably, GT-GRN embeddings exhibit tighter and more biologically coherent clusters that align with functional gene modules, suggesting that the model captures regulatory programs. These preserved clusters provide evidence of pathway-level organization, highlighting the ability of GT-GRN to reveal biologically meaningful communities relevant to cellular processes.

### GT-GRN for cell-type annotation

**Figure 8:**
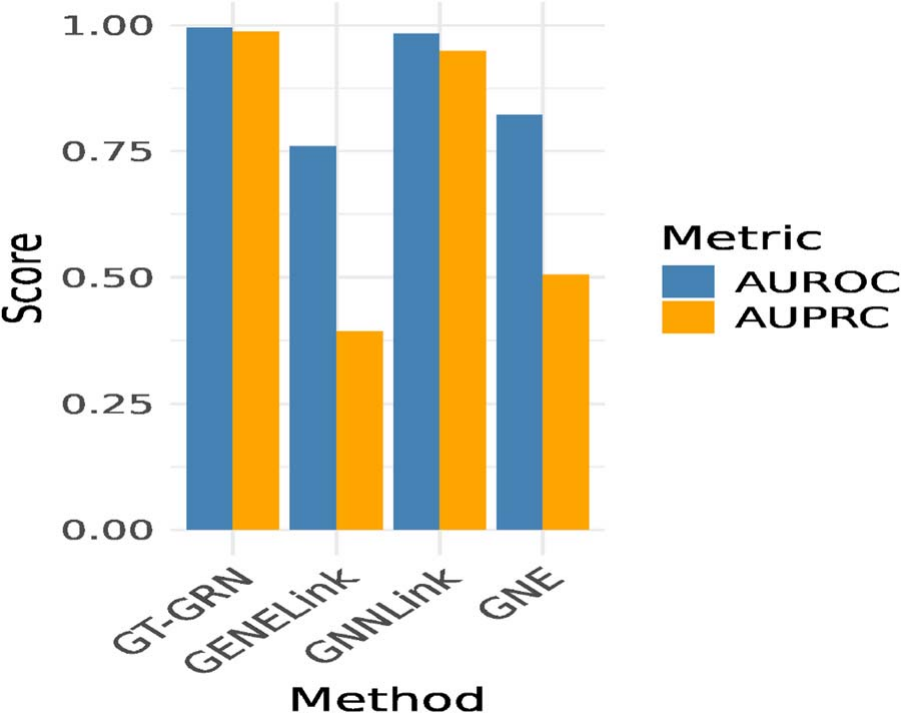
Cell-type classification. AUROC and AUPRC score of GT-GRN, GENELink, GNNLink, GNE-based embeddings in annotation cell-types using MLP classifier in five-fold cross-validation setting.

We further investigate these embeddings for cell-type annotation task. We manually annotate cell-types for gene expression data and focus on four cell-types with highest number of cells, *CD4+ T* cells, *CD14+ Monocyte cells*, and *CD8+ T cells*. We train a three-layered multilayered perceptron classifier for annotating cell-types. The classifier is trained using multiclass classification setting with five-fold cross validation. We benchmark GT-GRN against GENELink, GNNLink, and GNE methods. Figure 8 demonstrates that GT-GRN effectively captures the cell-types using gene representation classification setup.

Next, we delve into the individual contributions of each embedding modality within the GT-GRN framework through a systematic ablation study on the PBMC dataset. This dataset provides a biologically rich and diverse single-cell expression landscape, making it an ideal benchmark for evaluating the role of each embedding component in GRN inference.

### Ablation studies

To assess the overall efficacy and robustness of GT-GRN. We conducted the ablation study in two stages: first at the modality level, followed by the GT layer level. At the modality level, we systematically examined the contributions of structural positional encodings, global embeddings, and gene expression embeddings, both individually and in combination. Subsequently, we performed ablation experiments to assess the role of its internal components of GT layer. We first report the results of the modality-level study, and then present the findings from the GT layer ablation.

#### Modality-level ablation study

The modality-level ablation study systematically examines the individual and combined contributions of its embedding modalities: structural positional encodings, global embeddings, and gene expression embeddings. This experiment is crucial, given the multicomponent nature of our framework, as it allows us to systematically assess the role and efficacy of each component in the GRN inference process under a link prediction setup. We organize our baselines into three categories: uni-modal, bi-modal, and tri-modal, where the prefixes “uni,” “bi,” and “tri” denote the number of information sources used. This study establishes the necessity of each module in the overall architecture, demonstrating that omitting any single modality leads to a significant performance drop—thereby justifying the integration of all components for optimal GRN reconstruction.

- *Structural positional encodings*: In this uni-modal baseline, we have used the $PE_{graph}$ of the input network which is then fed into the GT-GRN. Here, each gene positional encodings is of length 512. This vector is fed into the model to predict possibility of link with other gene vectors based on the input link information.
- *Global embeddings*: This is a uni-model baseline, that extracts the global knowledge of inferred GRNs in a multinetwork integration framework using BERT model. The output length of each gene embedding here is 512. The vector is given as input into the model to estimate the likelihood of forming links with other gene vectors for link inference.
- *Gene expression embeddings*: In this uni-modal baseline, raw gene expression is converted into a embedding vector to capture the latent information using auto-encoder model. The length of the embedding vector for each gene is 512 which is then used to estimate the likelihood of the link using the GT-GRN framework.
- *Structural positional encodings + Global embeddings*: This multimodal approach combines the structural positional encodings of the input network with global embeddings derived from the BERT-based multinetwork integration framework. The fused representation is used in the GT-GRN model to enhance link prediction performance.
- *Structural positional encodings + Gene expression embeddings*: This approach integrates the structural positional encodings of the input network with gene expression embeddings obtained through an autoencoder. The combined vector representation is fed into the GT-GRN model to infer potential links between genes.
- *Global embeddings + Gene expression embeddings*
  In this setup, global embeddings capturing inferred GRN knowledge are combined with gene expression embeddings. The resulting representation is used to predict gene interactions within the GT-GRN framework.
- *Structural positional encodings + Global embeddings + Gene expression embeddings*: This comprehensive multimodal approach fuses all three embeddings—structural positional encodings, global embeddings, and gene expression embeddings—to provide a richer representation for link inference. This integrated approach aims to leverage complementary information from multiple modalities to enhance predictive performance.

Table 6 reports the ablation study results from different feature sets for GT-GRN using AUROC scores. Among unimodal representations, Global Embeddings achieve the highest AUROC of 0.8860, followed by gene expression embeddings (0.8693) and structural positional encodings with AUROC score of 0.8480, indicating that global information is the most informative for link inference.

**Table 6:** Ablation study in terms of different features for *PBMC’s* dataset for GT-GRN framework

Modality	Feature sets	AUROC
Unimodal	Structural positional encodings	0.8480
	Global embeddings	0.8860
	Gene expression embeddings	0.8693
Bimodal	Structural positional encodings + Global embeddings	0.8843
	Structural positional encodings + Gene expression embeddings	0.8666
	Global embeddings + Gene expression embeddings	0.8841
Trimodal	Structural positional encodings + Global embeddings + Gene expression embeddings	**0.9852**

The bold value indicates the best performance.

Bimodal combinations improve performance, with Global embeddings + Structural positional encodings (0.8843) and Global embeddings + Gene expression embeddings (0.8841) performing best. The trimodal combination of all three embeddings achieves the highest AUROC of 0.9852, demonstrating that integrating multiple modalities provides the most effective representation for GRN inference.

Overall, the study highlights the importance of multimodal integration, with Global Embeddings playing a key role in enhancing predictive performance.

#### Graph transformer layer ablation study

This study explores how different components of the GT layer impact the model’s performance, specifically in the context of predicting GRNs. The ablation study isolates specific components to assess their importance for the overall performance of the model. These components include attention heads, depth, FFN, normalization, and residual connections.

Table 7 presents the impact of different components of the GT layer on GRN inference performance. It summarizes various model variants, detailing the specific modifications made to each component, and reports the corresponding AUROC scores. This analysis highlights how changes to the GT layer architecture influence model effectiveness, providing insights into the relative importance of each component. Firstly, the full model demonstrates the highest performance, with an AUROC of 0.9852, emphasizing the importance of all components working in harmony to provide the most effective representation for GRN inference. When individual components, such as attention heads, FFN, or layer depth, are removed, the performance decreases, highlighting the critical role each element plays in the model’s effectiveness.

**Table 7:** Ablation study of GT Layer components

Model variant	Description of change	Metric (AUROC)
Full model	All components included	**0.9852**
Reduced heads	Head = 1	0.9741
Reduced depth	Layers = 2	0.9619
Feedforward network	Remove FFN	0.8815
Normalization	Disable normalization	0.8442
Residual connections	Remove skip-connections between layers	0.8436

The bold value indicates the best performance.

Among the various ablations, the most significant performance drops occur when the FFN is removed or normalization is disabled. This indicates that these components are especially vital for the success of the GT layer. In general, the ablation study underscores the substantial benefits of incorporating multiple attention heads, a deeper structure, a FFN, normalization, and residual connections within the GT layer. Each of these components improves the predictive capabilities of the model and their removal leads to a marked decline in performance. Therefore, to achieve optimal results in GRN inference, it is essential to retain all these components.

## Conclusion

In this work, we proposed a novel GRN inference framework, GT-GRN, which leverages GTs to infer regulatory links by incorporating graph-based techniques. Our approach begins by generating gene embeddings through an autoencoder. We then integrate prior network knowledge from known GRNs using an NLP-based BERT model, where these graphs are converted into sequences to extract contextual embeddings. Additionally, we incorporate graph positional information to enhance the inference process.

Through extensive experiments, GT-GRN demonstrates superior link prediction performance compared to baseline methods. We further assess the quality of the generated embeddings by evaluating their community structure, showing that GT-GRN effectively supports cell-type annotation in real PBMC gene expression datasets. Our ablation study reveals that the combination of gene expression data, global gene context, and positional information significantly contributes to improved GRN inference precision. While the current work focuses on accurate reconstruction of GRNs using multimodal embeddings, ongoing efforts are directed toward extending the framework to prioritize disease-associated genes. By leveraging the inferred GRNs, the goal is to identify key regulatory hubs and pathways that may play pivotal roles in disease development and progression.

**Key Points**

- GT-GRN is a novel graph transformer framework for enhanced gene regulatory network inference.
- It leverages multimodal embeddings by integrating gene expression data, prior biological network knowledge, and graph positional encodings.
- Proposed model outperforms baseline models, achieving higher predictive accuracy and robustness on various datasets.
- Achieves strong performance on cell-type classification using the peripheral blood mononuclear cell single-cell RNA sequence dataset and provides a scalable and extensible framework for diverse biological network analysis tasks.

## Supplementary Material

GT_GRN_FINAL_FINAL_SUPP_bbaf584

## Data Availability

All data and code used in this study are publicly available. The source code for *GT-GRN* can be accessed at https://github.com/Netralab/GT-GRN. The datasets used in our experiments are available at the following locations: https://zenodo.org/records/3701939 https://www.10xgenomics.com/datasets/8-k-pbm-cs-from-a-healthy-donor-2-standard-2-1-0
