# Supplementary material for: GT-GRN: a graph transformer framework for enhanced gene regulatory network inference via multimodal embedding of expression data and existing network knowledge
Source: Brief Bioinform. 2025 Nov 9;26(6):bbaf584. doi: 10.1093/bib/bbaf584 (PMC12597036; doi:10.1093/bib/bbaf584)
Supplement: GT_GRN_FINAL_FINAL_SUPP_bbaf584 [file gt_grn_final_final_supp_bbaf584.pdf]

Supplementary Materials for

GT-GRN: A Graph Transformer Framework for Enhanced Gene Regulatory Network Inference via Multimodal Embedding of Expression Data and Existing Network Knowledge

Binon Teji[1], Swarup Roy[1,2,*], Dinabandhu Bhandari[3], and Jugal Kalita[4]

[1]Network Reconstruction & Analysis (NetRA) Lab, Department of Computer Applications, Sikkim University, 6th Mile, Tadong, 737102, Sikkim, India
[2]Department of Computer Science & Engineering Tezpur University, Tezpur, Assam
[3]Department of Computer Science Engineering, Heritage Institute of Technology, Kolkata, 700107, West Bengal, India
[4]Department of Computer Science, University of Colorado, Colorado Springs, CO, USA
[*]Corresponding author: swarup@tezu.ernet.in

Supplementary Tables

Table T1: Runtime comparison of different methods.

Method	Runtime (h:m:s)
GT-GRN	1h 31m 26.53s
GNNLink	0h 11m 15.45s
GENELink	2h 14m 56.24s
GNE	0h 00m 39.12s

Supplementary Figures

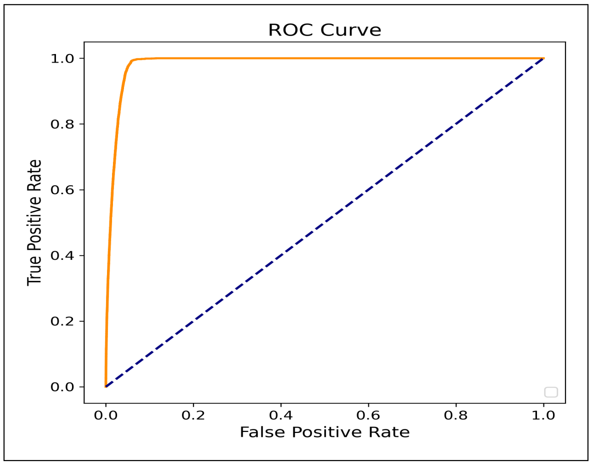

Figure S1: **ROC curve of the proposed GT-GRN model for PBMC dataset.**
Alt Text : *ROC curve of the* GT-GRN *model for the PBMC dataset.*

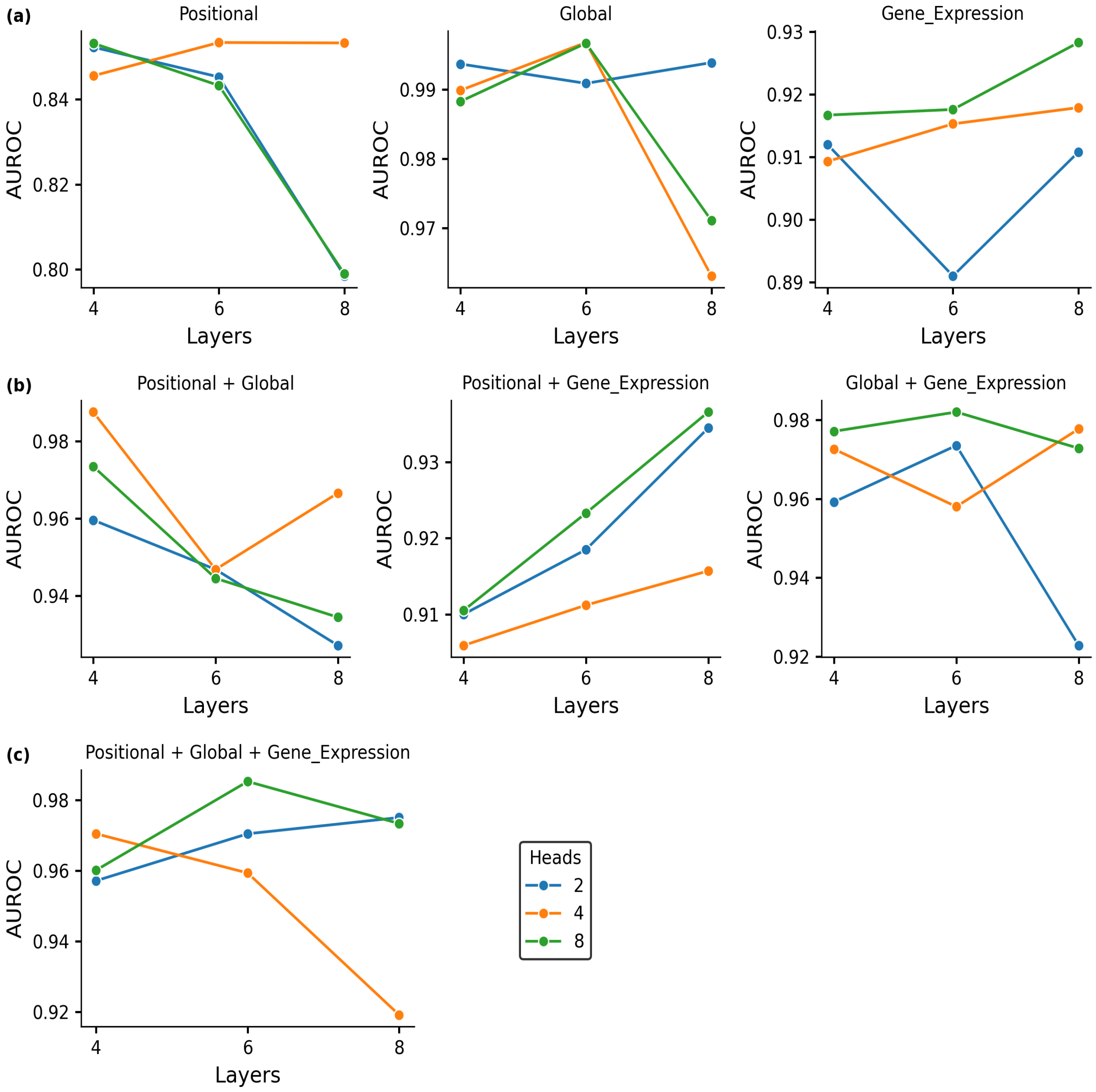

Figure S2: **Hyperparameter tuning results for the *GT-GRN* over PBMC dataset. Each subplot illustrates the effect of varying the number of layers (x-axis) and attention heads (legend) on AUROC (y-axis) for different combinations of embedding modalities**: (a) *single modalities — positional, global, and gene expression embeddings;* (b) *two-modal combinations — positional + global, positional + gene expression, and global + gene expression;* (c) *all three modalities combined — positional + global + gene expression.*
Alt Text : *Line plots showing AUROC versus number of layers for different embedding combinations and attention heads. The figure compares single, dual, and triple modality settings, illustrating how performance changes with model depth and attention heads.*
